# Nonword Repetition and Speech Motor Control in Children

**DOI:** 10.1155/2015/683279

**Published:** 2015-10-18

**Authors:** Christina Reuterskiöld, Maria I. Grigos

**Affiliations:** Department of Communicative Sciences and Disorders, New York University, 665 Broadway, Suite 922, New York, NY 10012, USA

## Abstract

This study examined how familiarity of word structures influenced articulatory control in children and adolescents during repetition of real words (RWs) and nonwords (NWs). A passive reflective marker system was used to track articulator movement. Measures of accuracy were obtained during repetition of RWs and NWs, and kinematic analysis of movement duration and variability was conducted. Participants showed greater consonant and vowel accuracy during RW than NW repetition. Jaw movement duration was longer in NWs compared to RWs across age groups, and younger children produced utterances with longer jaw movement duration compared to older children. Jaw movement variability was consistently greater during repetition of NWs than RWs in both groups of participants. The results indicate that increases in phonological short-term memory demands affect articulator movement. This effect is most pronounced in younger children. A range of skills may develop during childhood, which supports NW repetition skills.

## 1. Introduction

Language acquisition is often studied in isolation from neuromotor development [1, 2]. As a result, the relationship between higher-level language and cognitive skills and lower-level abilities related to speech output is poorly understood. There is a need to better understand this relationship not only from a theoretical standpoint but also from a clinical perspective as many diagnostic measures of language ability, such as nonword repetition, rely on speech production as a response mode. The purpose of the present study was to explore the interaction between cognitive/linguistic and speech motor processes by studying how children and adolescents modify articulatory control during the repetition of real words (RWs) and nonwords (NWs) that vary in length.

NW repetition is a task that has been widely used in assessment of children with language and literacy impairments and it has been suggested as a marker of the behavioral phenotype of Specific Language Impairment (SLI) [3, 4]. Children listen to pseudowords and are asked to repeat them as accurately as possible [5–9]. The notion underlying the use of NW repetition is that using unknown words (e.g.,* mustrefalj*) makes it difficult to access lexical knowledge in long-term memory to support performance. There is a large body of research demonstrating a link between NW repetition skills and language and literacy abilities in children and adolescents with and without language impairments (e.g., [10–16]).

NW repetition has been considered a measure of phonological short-term memory (PSTM) [8], but it is currently viewed as a task with high psycholinguistic complexity [11, 14, 17–19] taxing a range of input and output processes. The working memory model [20, 21] has been a widely used theoretical concept describing a limited capacity system supporting cognitive processes in children and adults. The model has been extensively used over the last few decades as a framework to explain behavior in terms of both development and disorders. The original theoretical model is comprised of three parts, the central executive and two slave systems. One of the slave systems is the phonological loop, which is responsible for short-term storage of recently presented unknown phonological information. The phonological loop is comprised of a storage unit, which retains phonological representations of language, and a subvocal rehearsal unit, which is a tool that aids in retention of novel phonological information [22]. According to Gathercole [11], in a short-term memory model there is a temporal decay of phonological representations. Longer stimuli will therefore be more vulnerable to decay than shorter stimuli due to the increased time of presentation and repetition [23]. The difference in performance between children with language impairment and children with typical development (TD) increases with length of the NWs [8, 24]. As Leonard [25] discusses, citing a meta-analysis by Estes and colleagues [26], there is much evidence that children with language impairment perform worse than children with TD also on shorter tokens even at a length of one syllable. This, according to Leonard [25], is evidence that processes other than PSTM contribute to the differences in performance between groups.

While it is obvious that speech motor processes are involved in NW repetition, it is not clear how different levels of articulation proficiency affect the formation of phonological representations. A few older studies have examined PSTM in individuals with severe motor speech disorders [27–29]. Such studies are interesting since they may help clarify whether articulation skills support phonological processing skills, including phonological memory. Bishop and colleagues studied individuals with anarthria (the inability to produce speech) who were diagnosed with cerebral palsy. The investigators showed that their participants were able to retain RWs long enough in memory to perform a judgment of same-different, but this was not the case when the stimuli were NWs. It was concluded that the retention of unfamiliar phonological word forms is supported by overt or covert articulation, a strategy that may not be available to individuals with speech impairment, which results in difficulty remembering NWs.

Although there appears to be a link between speech motor control and PSTM, few studies of NW repetition have also examined oral motor skills. In one of our earlier studies of five-year-old Swedish-speaking children with language impairment [14, 30], we found a correlation between NW repetition and expressive phonology, but not with performance on a test of oral motor skills. This result suggests that NW repetition taps into the representational level of phonology but is not linked to oral motor skills per se. More recently, Krishnan et al. [31] reported that differences in oral motor control in children contributed significantly to NW repetition scores independent of age and general language or cognitive skills. The authors suggested that poor oral motor control might be one of several risk factors for language impairment in children. The contrasting findings from these studies highlight the complex relationship between language and motor processing, which merits further investigation.

Developmental changes in articulatory control with respect to linguistic complexity are well documented. Typically, developing speakers modify spatial and temporal features of articulator movement throughout childhood and adolescence [32–40]. Maturational changes in lip and jaw movement include decreases in duration and increases in velocity [32, 33, 37, 39, 41]. Movement variability also decreases with age [32, 37, 42, 43]. Several studies have explored the influence of linguistic complexity on articulatory control. Maner et al. [44] examined lower lip movement changes in five- and eight-year-old children and adults during RW phrases that increased in length and syntactic complexity. The children consistently produced longer and more variable lower lip movements as utterances increased in length and complexity. Dromey and Benson [45] reported decreases in lip movement stability in adults when linguistic demands were placed on participants (i.e., verb generation during a sentence completion task). Further, they showed that speakers produced slower lip movements during taxing cognitive tasks (i.e., counting backward from 100 by 7). Walsh et al. [46] examined articulator movement in nine- to ten-year-olds and adults during the production of NWs that increased in syllable length. Both the adults and children showed a tendency for lip aperture variability, as well as lower lip/jaw variability, to increase as syllable length increased. Taken together, results from these studies demonstrate that speech motor control is influenced by cognitive and linguistic processing demands.

Several recent studies involving adult speakers have focused on the interaction between higher levels of processing and speech output [47–50]. McMillan et al. [47] investigated elicited slips of the tongue where participants were asked to repeat word pairs, which appeared on a screen for a brief period of time. In some of the word-pair cases subjects were cued to repeat the words in reversed order (*tum gop* resulting in* gop tum*). The outcome of the study showed that substitutions were more likely to occur when RW competitors were present. Further, electropalatographic measures revealed greater variability when stimuli pairs consisted of NWs only. From these findings, the researchers concluded that there is a clear lexical bias in articulation and a relationship between cognitive and motor movements involved in speech processing and production [47]. To tap into this relationship in children, Heisler et al. [51] examined the influence of word learning on speech production during a novel word-learning task. They compared phonetic accuracy and movement pattern stability during the production of phonetic forms with and without lexical representation (a visual referent and/or object function). The results showed that production of a novel phonetic sequence was less variable in terms of articulatory movement when paired with a visual and/or functional lexical referent.

There is a need for research that can lead to a deeper understanding of the perceptual, cognitive, and motor processes that are involved in NW repetition, as well as their relationship with linguistic processes [52–56]. The focus of the current work is to examine the influence of increased PSTM demands (repetition of RWs versus NWs) on speech motor control in typically developing children and adolescents. We explored the hypothesis that articulator movement duration and variability will increase during tasks with greater PSTM demands. Moreover, we investigated whether there are age-related differences in articulator movements related to word type, hypothesizing that differences between RWs and NWs would be larger in children than in adolescents. Specific questions guiding this study were as follows. (1) Does jaw movement duration and stability differ between children and adolescents during the repetition of RWs versus NWs with similar phonetic properties? (2) Is articulatory control for the production of RWs versus NWs influenced by increases in stimuli length in children and adolescents?

## 2. Method

### 2.1. Participants

Sixteen participants completed the study and were categorized into either a younger or older age group (eight participants per group (four males/four females)). Mean age (standard deviation) was 6.10 (1.6) in the younger group and 14.4 (1.8) in the older group. These age groupings were selected to compare the effects of increased cognitive demands on articulator movements between children and adolescents. It is well documented that articulatory control differs between children and adolescents [40] and that children's performance on working memory measures improves with age [57]. It is not clear, however, how the interaction between cognitive and speech motor skills changes with increased age. All participants were monolingual speakers of American English, with no reported histories of speech, language, hearing problems, or neurological disorders. The study was approved by Institutional Review Board at New York University and informed consent was obtained from all participants and their parents.

Speech and language skills were formally and informally assessed. Speech production skills were examined using the Goldman Fristoe Test of Articulation-2 (GFTA-2) [58] and through a conversational speech sample. Receptive and expressive language skills were evaluated using the Clinical Evaluation of Language Fundamentals (CELF-P; CELF-3) [59, 60]. The Verbal Motor Production Assessment in Children (VMPAC) [61] was used to examine oral motor skills. The participants demonstrated age appropriate speech, language, and oral motor skills on these measures. All participants passed a pure-tone hearing screening presented bilaterally at 25 dB at .5, 1, 2, and 4 kHz.

In order to rule out any major difficulties with NW repetition, we included a NW repetition task in our pretest procedure. NW repetition was assessed using the Children's Nonword Repetition Test (CNrep) [57]. NWs were recorded by a speaker of American English and the task was completed in a sound treated booth. Participants were instructed to listen to and repeat each NW presented through speakers. Responses were recorded and percent consonants correct (PCC) [62] scores were calculated separately by two trained graduate students in speech language pathology. Mean PCC for the repeated targets in the CNrep test were 88.88% for younger group and 98.1% for the older group.

### 2.2. Signal Recording and Processing

Jaw movement was tracked in three dimensions using a motion capture system, Vicon 460 [63]. Ten reflective markers (each 3 mm in diameter) were placed on the face. Five markers were used to track lip and jaw movement and were placed on the midline of the vermilion border of the upper lip, midline of the vermilion border of the lower lip, superior to the mental protuberance of the mandible, and on the corners of the mouth. Five reference markers were used to account for head movement and rotation, which were placed on the nose, nasion, and forehead. Jaw movement was calculated by subtracting *y* coordinates from the stationary points on the forehead.

Kinematic data analysis was conducted using MATLAB, version 7.2 [64]. The system tracked reflective markers at a sampling rate of 120 frames per second. Audio recordings were made using a digital minidisc recorder, M-Audio, MicroTrack 2496. Participants wore a lapel microphone, Audio-Technica, Model AT831W, which was placed on the shirt approximately 6 inches from the mouth. All recordings were made in a sound attenuated audiometric booth at New York University.

### 2.3. Data Collection and Procedures

Participants listened to recordings of a monolingual American-English-speaking adult producing RWs and NWs. They were told that they would be hearing “real words” and “funny, made-up words” and were asked to repeat the structures exactly as they heard them using their habitual speaking rate and loudness. Referents were not provided for the RWs or NWs. Eight practice items (four RWs and four NWs) were administered. If a participant requested additional practice items or if the experimenters felt that they did not completely understand the task, the practice items were repeated. This occurred in two of the younger participants, who did not have any difficulty completing the experimental protocol after additional practice. The tokens included two RWs (i.e., “baby muppet”/bebi mʌpɪt/ and “peppy mama muppet”/p*ε*pi mam*ə* mʌpɪt/) and two NWs (i.e., “babu mepid”/bᴂb*ə* m*ε*pɪd/ and “bebu pupu bepid”/bɛb*ə* pʌp*ə* bɛpɪd/), which were presented in a randomized order in terms of word type (RW versus NW) and length (four versus six syllables). By the end of the session, fifteen productions of each token were obtained from the subjects. These RW and NW structures were selected because they include bilabial phonemes, /p/, /b/, and /m/, that allowed lip and jaw movements to be visualized. NWs did not contain any syllables that constituted real words. RWs and NWs were matched in number of syllables, stress pattern, linguistic complexity, and phonotactic probability (Table 1) [14, 65–67]. The latter is an index of the probability of a segment occurring in combination with one or two other segments in the sequential arrangement in the word. A higher value means a higher probability of occurrence or the combinations of segments included in the targets.

### 2.4. Analyses

#### 2.4.1. Perceptual Judgments

A graduate student in speech language pathology, naïve to the purpose of the experiment, listened to and transcribed all of the productions of each token from each speaker. A second graduate student transcribed 10% of speaker productions from randomly chosen participants. Interrater agreement on PCC scores was computed on a segment to segment basis and reached 99%. The perceptual analyses included measures of percent consonant correct (PCC) [62] and percent vowel correct (PVC). PCC and PVC were calculated separately for each participant (1) for the first production of each token (total of 64 utterances: 4 tokens × 1 production × 16 participants) and (2) across all productions of each token (total of 960 utterances: 4 tokens × 15 productions × 16 participants). The rationale for examining first productions was to calculate segmental accuracy of the productions of each NW the first time they were produced. Segmental accuracy was also examined across all productions to obtain a more comprehensive index of articulation performance.

#### 2.4.2. Kinematic Analysis

The kinematic analysis was based upon accurate productions identified through the perceptual analysis. Only accurate productions were included to ensure that any observed kinematic differences are due to underlying changes in speech motor control that are independent of articulation errors. Given the high variability of children's articulator movements, multiple productions of the same token from each child were analyzed, rather than also examining first productions of tokens. The first eight productions, in which segmental and suprasegmental components were judged to be accurate and in which all reflective markers were visible, were included in the analyses. Eight productions were selected as this was the greatest number of productions across all participants that met the criteria mentioned above. Productions were eliminated due to one or more of the following factors: consonant/vowel error, suprasegmental error (e.g., equal stress), and missing reflective markers. Segmental and suprasegmental errors were more prevalent in the younger children. A total of 512 utterances were included in the kinematic analyses (4 targets × 8 productions × 16 participants).

**Figure 1:**
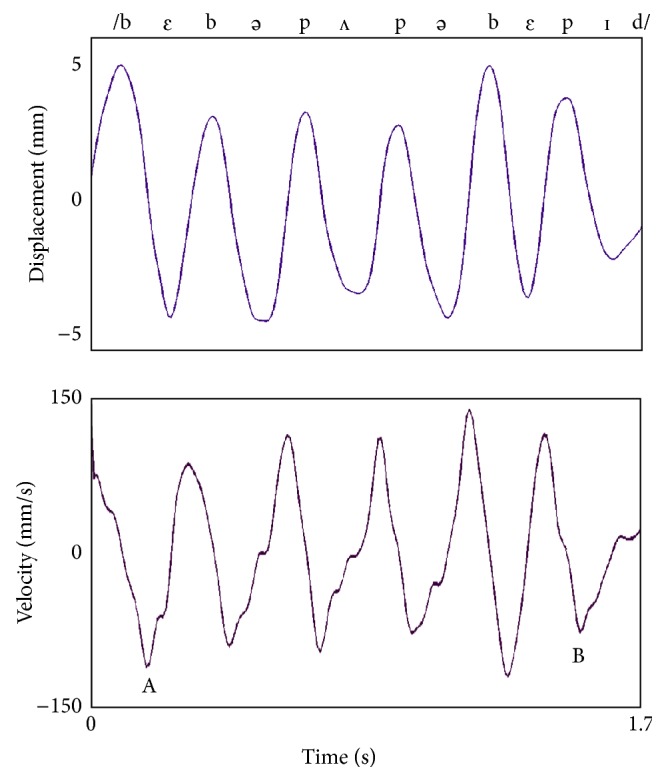
Kinematic traces of jaw velocity and jaw displacement corresponding to the utterance /bɛb*ə* pʌp*ə* bɛpɪd/. Duration measures are based upon velocity points. Total movement duration is calculated as the time between movement onset and movement offset. Movement onset is the velocity minima associated with oral opening for the first vowel in the word (i.e., /ɛ/) and movement offset is the velocity minima associated with oral opening for the final vowel in the word (i.e., /ɪ/) (points A to B).

The acoustic and kinematic signals from each production were aligned. The acoustic signal was used to help identify articulator movement associated with each word. Kinematic records of the jaw were then analyzed. The onset and offset of movement were based upon velocity minima in the jaw kinematic trace. The onset of movement was selected as the point of minimum velocity into oral opening for the first vowel in the word. The point of minimum velocity into opening for the word final vowel was chosen as the movement offset. Total movement duration was calculated as the time between movement onset and movement offset in the jaw velocity trajectory (Figure 1).

Movement trajectory stability was examined to explore whether there are changes in stability of the underlying movement pattern associated with the production of real words and nonwords across development once differences in absolute time and amplitude were removed. The onset and offset of movement identified in the velocity trace for the total duration measure was also used to segment displacement data for the movement stability analysis. Segmented displacement traces were normalized for amplitude and time. For each displacement trace, amplitude normalization was achieved by subtracting the mean of the displacement record and dividing by its standard deviation. Time normalization was achieved by using a cubic spline procedure to interpolate each waveform onto a time base of 1,000 points. The spatiotemporal index (STI) was then calculated to examine stability in movement trajectories across repeated productions of target utterances [68]. The STI was computed by calculating standard deviations at 2% intervals across repetitions of the time and amplitude normalized displacement traces. The STI is the cumulative sum of these 50 standard deviations. The STI indicates the degree to which the set of trajectories converge onto one fundamental movement pattern [69].

#### 2.4.3. Statistical Analyses

Means and standard deviations were calculated for PCC, PVC, jaw movement duration (DUR), and STI, for each participant. Repeated measures analyses of variance (ANOVAs) were performed to examine the effects of the between-subjects variable* Group* (younger or older) and the within-subjects variables* Word Type* (RW or NW) and* Length* (four or six syllables) on PCC, PVC, DUR, and STI. Interactions between* Word Type*,* Length*, and* Group* were also measured. Each dependent measure was examined separately. When the main effect of* Word Type* was significant, pairwise contrasts were performed to explore differences between RWs and NWs within each experimental group. A Bonferroni correction factor was used to account for multiple comparisons within each variable (RW versus NW in the younger group and in the older group), which adjusted the alpha level to 0.025.

## 3. Results

### 3.1. Perceptual Accuracy

PCC and PVC scores for each RW and NW for first repetitions, as well as across all repetitions for each participant, are shown in Table 2.

### 3.2. First Productions

Comparisons of first productions revealed a trend of greater consonant and vowel accuracy in RWs than NWs. Consonant and vowel accuracy were higher for RWs than NWs as evidenced by a significant main effect of* Word Type* on PCC, *F*(1,14) = 13.75, *p* = 0.002, *η*_*p*_^2^ = 0.495, and PVC, *F*(1,14) = 9.95, *p* = 0.007, *η*_*p*_^2^ = 0.415. PCCs and PVCs were similar between four- and six-syllable structures and between experimental groups. Thus, there were no significant main effects of* Length* or* Group* on PCC or PVC. Further, there were no significant interactions between* Word Type*,* Length*, and* Group*.

#### 3.2.1. All Productions

When all productions were examined, consonant accuracy was significantly higher in the RWs as compared to the NWs. This observation was supported by a significant main effect of* Word Type* on PCC, *F*(1,14) = 18.283, *p* = 0.001, *η*_*p*_^2^ = 0.574. There were no significant main effects of* Length* or* Group* on PCC as consonant accuracy was similar between four- and six-syllable tokens for both groups. There were no significant two- or three-way interactions between* Word Type*,* Length*, and* Group*.

All participants produced vowels with greater accuracy in RWs than in NWs. This finding was supported by a significant main effect of* Word Type* on PVC, *F*(1,14) = 10.45, *p* = 0.006, *η*_*p*_^2^ = 0.427. The difference in vowel accuracy between RWs and NWs was evident in both four- and six-syllable structures, as well as in the younger and older groups. Thus, there were no significant main effects of* Group* or* Length*. Interactions between* Word Type*,* Length,* and* Group* were not significant.

### 3.3. Articulator Movement

Total jaw movement duration (DUR) and movement stability (STI) were calculated from all accurate productions of RWs and NWs.

#### 3.3.1. Movement Duration

**Figure 2:**
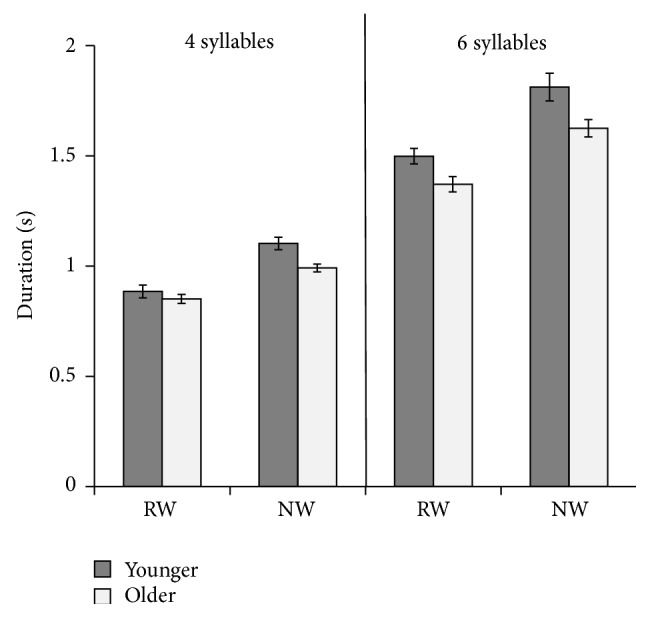
Mean total jaw movement duration and standard error in the younger and older groups producing four-syllable (left column) and six-syllable (right column) real words (RWs) and nonwords (NWs).

Jaw movement duration was longer in NWs than RWs during the production of four- and six-syllable structures across both groups (Figure 2). As expected, movement duration was longer for six- than four-syllable structures. These findings were supported by significant main effects of* Word Type*, *F*(1,14) = 79.49, *p* < 0.001, *η*_*p*_^2^ = 0.850, and* Length*, *F*(1,14) = 1082.32, *p* < 0.001, *η*_*p*_^2^ = 0.987, on jaw movement duration. There was also a significant main effect of* Group* on duration, *F*(1,14) = 11.95, *p* = 0.004, *η*_*p*_^2^ = 0.460. Participants in the younger group produced utterances with significantly longer jaw movement durations as compared to the older group. A significant interaction between* Length* and* Group* was found, *F*(1,14) = 5.04, *p* = 0.041, *η*_*p*_^2^ = 0.265, where the difference in duration between four- and six-syllable tokens was greater in the younger than in the older group. Further, the interaction between* Length* and* Word Type* was significant, *F*(1,14) = 6.24, *p* = 0.026, *η*_*p*_^2^ = 0.308, as differences between NWs and RWs were larger in the six-syllable than in the four-syllable tokens. Post hoc tests revealed significantly longer movement duration in NWs than RWs in both the younger group (mean difference = 0.265, *p* < 0.001) and the older group (mean difference = 0.198, *p* < 0.001). A three-way interaction between* Word Type*,* Length,* and* Group* was not significant.

#### 3.3.2. Movement Stability

**Figure 3:**
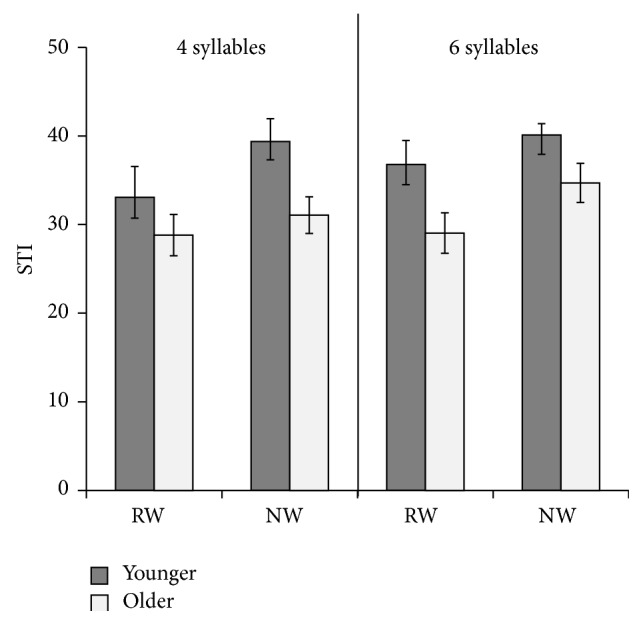
Mean jaw STI and standard error in the younger and older groups producing four-syllable (left column) and six-syllable (right column) real words (RWs) and nonwords (NWs).

Comparisons of spatiotemporal stability were performed by examining changes in the jaw STI across age groups in four- and six-syllable RWs and NWs. High STIs indicate greater spatiotemporal variability and low STIs represent more stability across movement trajectories. STIs were higher in NWs than RWs in both groups (Figure 3). This finding was supported by a significant main effect of* Word Type* on jaw STI, *F*(1,14) = 8.20, *p* = 0.013, *η*_*p*_^2^ = 0.369. There was no main effect of* Length* on STI as movement stability was similar between four- and six-syllable structures with each word type. Group effects were evident, however, as STIs were higher in the younger than the older participants, *F*(1,14) = 5.75, *p* = 0.031, *η*_*p*_^2^ = 0.291. There were no significant interactions between* Word Type*,* Length*, and* Group*.

## 4. Discussion

Our main interest was to determine whether speech motor patterns of children and adolescents were equally vulnerable to increases in cognitive demands. Specifically, we examined patterns of movement duration and variability across multiple accurate productions of RWs and NWs. Our findings illustrate that both jaw movement duration and stability differed between RWs and NWs, although to varying degrees in the children and adolescents based on word length. These results support the hypothesis that articulator movement duration and variability will increase during tasks with greater PSTM demands.

### 4.1. Nonword Repetition Accuracy

Accuracy of production has been the traditional measure of NW repetition and considered an index of phonological short-term memory skills provided one production only was permitted in order to avoid a practice effect. In the present work, first productions were found to have higher consonant and vowel accuracy in RWs than in NWs across word lengths and groups, as expected. These results are consistent with numerous studies showing that accuracy scores are higher during repetition of RWs compared with NWs in children with and without language impairment [8, 14, 19]. Our results showing similar consonant accuracy between younger and older children did not correspond with results from past research, which reported increased NW repetition accuracy with age [4, 12]. These researchers viewed a developmental trend as evidence that PSTM skills continue to develop with increased age during childhood and adolescence. A careful inspection of the data in Table 2 illustrates a trend for the differences between RWs and NWs to be greater in the younger than the older children for the four-syllable tokens yet similar between the six-syllable tokens. Thus, it is plausible that our findings may have more closely mirrored those from earlier studies if a larger participant pool and more complex NWs were examined. These limitations are discussed in greater detail below.

Differences between RW and NW accuracy that were seen during first productions were also evident when all productions were analyzed. Across all productions, consonant accuracy remained higher in RWs compared to NWs in both groups. Vowel accuracy was also higher in RWs than NWs in both groups of children. Overall, the consistency of accuracy between first productions and all productions suggests a sustained effect of a more cognitively challenging production task. Nonetheless, both groups of participants were able to achieve many accurate productions of both the RWs and NWs.

### 4.2. Influence of Word Type on Articulator Movement

Jaw movement duration was significantly longer in NWs compared to RWs across both word lengths and age groups, illustrating that all participants were taxed by NW production and increased movement duration to complete this task. These results suggest that both children and adolescents may compensate for the increase in cognitive demand by modifying the temporal control of speech movements. Our findings also support past research that has shown that articulatory control follows a protracted course of development [40, 43].

Age-related differences in temporal control influenced patterns seen between the younger and older children. The older children consistently produced both RWs and NWs with shorter movement durations than the younger children, which is consistent with past research showing that movement duration decreases with age [32, 33, 37, 39, 41]. Duration differences between RWs and NWs in both the four- and six-syllable tokens were significantly greater in the younger than in the older children. This suggests that the articulatory patterns of the younger children were more vulnerable to task demands. The significant interaction between* Length* and* Word Type* resulted from the differences between RWs and NWs being greater in the six-syllable tokens than in the four-syllable tokens, a finding that was more pronounced in the younger than the older participants. Taken together, these results illustrate that more mature temporal control seen in the older as compared to the younger group may facilitate greater consonant and vowel accuracy during the NW task. These findings are consistent with evidence that children modify temporal control during other speaking tasks, such as marking linguistic [37] and prosodic contrasts [35, 36].

Jaw movement variability, as measured by the spatiotemporal index (STI), was also examined across multiple accurate productions of tokens. A high spatiotemporal index (STI) indicates more jaw movement variability across productions. Movement variability was higher in the younger than older group for both NWs and RWs. Comparisons by* Word Type*, however, revealed that movement variability was greater during repetition of NWs compared with repetition of RWs in both groups of children. Thus, both the younger and older participants were challenged by the NWs even though they achieved perceptually accurate productions of the NWs. This result suggests that developing speakers may continue to alter speech motor planning and execution processes to meet the cognitively taxing demands of a NW task even during adolescence. Greater variability in the productions of NWs may be attributed to their less mature speech motor skills. An alternate explanation is that greater variability may reflect more movement flexibility required for children and adolescents to achieve accurate NW production. Taken together, these results are consistent with past research showing that articulatory control becomes more stable with maturation [32, 37, 42, 43] and continues to stabilize into adolescence [40].

It is important to highlight that differences in movement patterns observed between RWs and NWs were seen even though consonant and vowel accuracy were at 100% for these tokens. This suggests that demands on PSTM influence articulator movement although changes may not lead to perceptually detectable differences. This finding is particularly interesting given that the NW tokens were relatively simple in phonetic make-up. This observation leads us to speculate that there may be differences in children and adolescents pertaining to how the system reorganizes itself in order to complete more challenging speech production tasks. Such differences may lead to trade-off effects between linguistic processing and speech motor control that are measurable although not necessarily perceptually detectable. Just as others have suggested that there are trade-offs between linguistic levels during language production [70–74], the current findings suggest reciprocity between linguistic processing and speech motor control in children and adolescents.

Together, these findings suggest that when PSTM is taxed, children and adolescents compensate differently in order to achieve accurate word production. Both groups modified temporal control and movement variability to achieve accurate productions of RW and NW tokens. Our results showing that task demands influence articulatory control even during adolescence are not surprising as several studies have reported that adults are also sensitive to increases in cognitive demands during speaking tasks [45, 46]. It could be argued that examining articulatory control across repeated productions of NWs (rather than first productions of NWs) is a practice exercise rather than a word repetition task. We examined results from first productions in terms of accuracy but, in order to obtain measures from articulatory patterns, multiple productions were necessary. Our results show differences between articulatory patterns during production of RWs versus NWs in spite of multiple productions indicating an effect of cognitive load on speech motor control in spite of a possible practice effect.

### 4.3. Subskills Involved in Nonword Repetition

Factors, such as subvocal rehearsal and vocabulary growth, have been proposed to play a role in NW repetition. According to Gathercole [11], subvocal rehearsal, examined during first productions of a novel target, is not typically present in children younger than seven years. We found a significant difference in accuracy of first productions of RWs versus NWs but no significant differences between the age groups. This result indicates that both groups may have used this strategy to support PSTM, which might be expected since the mean age of our younger group was almost seven.

NW repetition is a complex psycholinguistic task and there is more to be learned about the contributing processes involved in performance in speakers across ages and clinical categories. Our results lend support to the view that there is a mutually dependent relationship between speech motor processes and the lexical level of processing of a target for production. It is interesting to observe that even when targets are well matched and are not containing phoneme combinations of high complexity or low probability, word type seems to affect children's accuracy of production and the processes involved in articulatory control. Adams and Gathercole [75] stated that output processes should not be used to “explain away” the relationship between phonological memory and language development. The contribution of speech motor processes to NW repetition skills in children is an area, which needs to be further investigated, however.

### 4.4. Methodological Limitations

Findings of the present work may be influenced by the construction of RW and NW targets. Although construction of NWs is known to be challenging (for a discussion, see [14, 65]), we made every attempt to match the RWs and NWs by syllable number, phonetic make-up, and phonotactic probability. Our longest structures were six syllables long but did not contain any clusters or phoneme combinations with low phonotactic probability. These factors have been described in the literature to tax NW repetition skills [7, 76]. Edwards and colleagues [77] reported higher accuracy in NW repetition for high frequency phonological patterns compared with low-frequency patterns in children. This effect decreased with increasing age, a result the authors attributed to larger vocabularies in the older children.

A second issue that should be pointed out is that construction of NWs matched to a selection of RWs known to young children and suitable for the kinematic procedures proved to be a significant challenge. We avoided syllables consisting of RWs when constructing our NWs. Further, bilabials were used in the tokens because facial tracking technology records movements from visual structures, such as the lips and jaw, which are involved in bilabial production. Using movement tracking was advantageous as this was a direct approach to investigating output processes involved in NW repetition. In comparison, many previous studies have employed indirect approaches, such as speed of articulation (i.e., speech rate) in producing sets of words [8, 22, 78, 79] or correlations between accuracy measures and results on oral motor tasks [14, 31].

## 5. Conclusion

In the present study, children and adolescents showed a lower level of consonant and vowel accuracy during NW repetition compared with repetition of RWs. Jaw movement duration was longer and variability of articulator movements was greater in NWs compared to RWs. Young children showed longer duration of jaw movements than adolescents. It is possible that a range of skills develop between the ages of six and fourteen, which support NW repetition skills. NW repetition is a complex task requiring a range of subskills. Future studies should continue to examine the role of less studied skills supporting NW repetition in children, such as speech motor control and orthographic skills. In addition, changes in articulator movement related to practice and learning during production of novel items should be further investigated.

## Figures and Tables

**Table 1:** Phonotactic probability.

	Real words	Nonwords
PAIR 1		
Phonotactic probability	0.44	0.42
Biphonotactic probability	0.01	0.02
PAIR 2		
Phonotactic probability	0.36	0.37
Biphonotactic probability	0.01	0.3
PAIR 3		
Phonotactic probability	0.44	0.35
Biphonotactic probability	0.01	0.02
PAIR 4		
Phonotactic probability	0.55	0.44
Biphonotactic probability	0.02	0.03

**Table 2:** Percent consonant correct (PCC) and percent vowel correct (PVC) from first repetitions and all repetitions.

Group	First repetition				All repetitions				First repetition				All repetitions			
	4-syllable RW		4-syllable NW		4-syllable RW		4-syllable NW		6-syllable RW		6-syllable NW		6-syllable RW		6-syllable NW	
	/bebi mʌpɪt/		/bᴂbə mɛpɪd/		/bebi mʌpɪt/		/bᴂbə mɛpɪd/		/pɛpi mamə mʌpɪt/		/bɛbə pʌpə bɛpɪd/		/pɛpi mamə mʌpɪt/		/bɛbə pʌpə bɛpɪd/	
	PCC	PVC	PCC	PVC	PCC	PVC	PCC	PVC	PCC	PVC	PCC	PVC	PCC	PVC	PCC	PVC
Younger																
S1	100.0	100.0	80.0	100.0	100.0	100.0	80.0	100.0	86.0	67.0	86.0	67.0	100.0	83.0	100.0	100.0
S2	100.0	100.0	80.0	100.0	100.0	100.0	60.0	100.0	100.0	83.0	86.0	67.0	100.0	100.0	71.0	63.0
S3	100.0	100.0	60.0	75.0	100.0	100.0	100.0	100.0	86.0	100.0	86.0	100.0	100.0	100.0	100.0	100.0
S4	60.0	100.0	80.0	100.0	60.0	100.0	14.0	67.0	86.0	100.0	57.0	83.0	71.0	100.0	60.0	75.0
S5	100.0	100.0	100.0	100.0	100.0	100.0	100.0	100.0	100.0	100.0	100.0	100.0	100.0	100.0	100.0	100.0
S6	100.0	100.0	60.0	100.0	86.0	100.0	80.0	100.0	100.0	100.0	28.0	100.0	100.0	100.0	29.0	83.0
S7	100.0	100.0	80.0	100.0	100.0	100.0	80.0	100.0	100.0	100.0	86.0	100.0	100.0	100.0	86.0	100.0
S8	100.0	100.0	100.0	100.0	100.0	100.0	100.0	100.0	100.0	100.0	100.0	100.0	100.0	100.0	74.0	85.0
Mean	**95.0**	**100.0**	**80.0**	**96.9**	**93.3**	**100.0**	**76.8**	**95.9**	**94.8**	**93.8**	**78.6**	**89.6**	**96.4**	**97.9**	**77.5**	**88.3**
SD	**14.1**	**0.0**	**15.1**	**8.8**	**14.3**	**0.0**	**29.0**	**11.7**	**7.2**	**12.3**	**24.4**	**15.1**	**10.3**	**6.0**	**24.8**	**14.2**
Older																
S9	100.0	100.0	100.0	100.0	100.0	100.0	80.0	100.0	100.0	100.0	100.0	100.0	100.0	100.0	86.0	83.0
S10	100.0	100.0	80.0	100.0	100.0	100.0	100.0	100.0	100.0	100.0	71.0	100.0	100.0	100.0	100.0	67.0
S11	100.0	100.0	100.0	100.0	100.0	100.0	80.0	75.0	100.0	100.0	43.0	67.0	100.0	100.0	57.0	100.0
S12	100.0	100.0	80.0	100.0	100.0	100.0	80.0	100.0	100.0	100.0	100.0	100.0	100.0	100.0	100.0	100.0
S13	100.0	100.0	100.0	100.0	100.0	100.0	100.0	100.0	100.0	100.0	100.0	83.0	100.0	100.0	100.0	83.0
S14	100.0	100.0	80.0	75.0	100.0	100.0	80.0	75.0	100.0	100.0	100.0	100.0	100.0	100.0	86.0	100.0
S15	100.0	100.0	100.0	100.0	100.0	100.0	100.0	100.0	100.0	100.0	86.0	83.0	100.0	100.0	86.0	83.0
S16	100.0	100.0	100.0	100.0	100.0	100.0	100.0	100.0	100.0	100.0	71.0	100.0	100.0	100.0	87.0	100.0
Mean	**100.0**	**100.0**	**92.5**	**96.9**	**100.0**	**100.0**	**90.0**	**93.8**	**100.0**	**100.0**	**83.9**	**91.6**	**100.0**	**100.0**	**87.8**	**89.5**
SD	**0.0**	**0.0**	**10.4**	**8.8**	**0.0**	**0.0**	**10.7**	**11.6**	**0.0**	**0.0**	**20.9**	**12.6**	**0.0**	**0.0**	**14.2**	**12.4**

## References

[1] Iverson J. M. (2010). Developing language in a developing body: the relationship between motor development and language development. *Journal of Child Language*.

[2] Iverson J. M., Braddock B. A. (2011). Gesture and motor skill in relation to language in children with language impairment. *Journal of Speech, Language, and Hearing Research*.

[3] Bishop D. V. M., North T., Donlan C. (1996). Nonword repetition as a behavioural marker for inherited language impairment: evidence from a twin study. *Journal of Child Psychology and Psychiatry and Allied Disciplines*.

[4] Conti-Ramsden G., Simkin Z. (2001). Non-word repetition and grammatical morphology: normative data for children in their final year of primary school. *International Journal of Language & Communication Disorders*.

[5] Kamhi A. G., Catts H. W. (1986). Toward an understanding of developmental language and reading disorders. *The Journal of Speech and Hearing Disorders*.

[6] Kamhi A. G., Catts H. W., Mauer D., Apel K., Gentry B. F. (1988). Phonological and spatial processing abilities in language- and reading-impaired children. *Journal of Speech and Hearing Disorders*.

[7] Gathercole S. E., Baddeley A. D. (1990). The role of phonological memory in vocabulary acquisition: a study of young children learning new names. *The British Journal of Psychology*.

[8] Gathercole S. E., Baddeley A. D. (1990). Phonological memory deficits in language disordered children: is there a causal connection?. *Journal of Memory and Language*.

[9] Gathercole S. E., Baddeley A. D. (1993). Phonological working memory: a critical building block for reading development and vocabulary acquisition?. *European Journal of Psychology of Education*.

[10] Catts H. W., Adlof S. M., Hogan T. P., Weismer S. E. (2005). Are specific language impairment and dyslexia distinct disorders?. *Journal of Speech, Language, and Hearing Research*.

[11] Gathercole S. E. (2006). Nonword repetition and word learning: the nature of the relationship. *Applied Psycholinguistics*.

[12] Gathercole S. E., Willis C. S., Emslie H., Baddeley A. D. (1992). Phonological Memory and Vocabulary Development During the Early School Years: A Longitudinal Study. *Developmental Psychology*.

[13] Montgomery J. W. (1995). Sentence comprehension in children with specific language impairment: the role of phonological working memory. *Journal of Speech and Hearing Research*.

[14] Sahlén B., Reuterskiöld-Wagner C., Nettelbladt U., Radeborg K. (1999). Non-word repetition in children with language impairment—pitfalls and possibilities. *International Journal of Language and Communication Disorders*.

[15] Sahlén B., Reuterskiöld-Wagner C., Nettelbladt U., Radeborg K. (1999). Language comprehension and non-word repetition in children with language impairment. *Clinical Linguistics and Phonetics*.

[16] Snowling M. J. (1981). Phonemic deficits in developmental dyslexia. *Psychological Research*.

[17] Montgomery J. W. (2003). Working memory and comprehension in children with specific language impairment: what we know so far. *Journal of Communication Disorders*.

[18] Whitehouse A. J. O., Barry J. G., Bishop D. V. M. (2008). Further defining the language impairment of autism: is there a specific language impairment subtype?. *Journal of Communication Disorders*.

[19] Montgomery J. W., Magimairaj B. M., Finney M. C. (2010). Working memory and specific language impairment: an update on the relation and perspectives on assessment and treatment. *American Journal of Speech-Language Pathology*.

[20] Baddeley A. D. (1986). *Working Memory*.

[21] Baddeley A. D., Hitch G. J., Bower G. (1974). Working memory. *The Psychology of Learning and Motivation*.

[22] Gathercole S. E., Adams A.-M., Hitch G. J. (1994). Do young children rehearse? An individual-differences analysis. *Memory and Cognition*.

[23] Baddeley A. D., Thomson N., Buchanan M. (1975). Word length and the structure of short-term memory. *Journal of Verbal Learning and Verbal Behavior*.

[24] Marton K. S., Schwartz R. G. (2003). Working memory capacity and language processes in children with specific language impairment. *Journal of Speech, Language, and Hearing Research*.

[25] Leonard L. (2014). *Children with Specific Language Impairment*.

[26] Estes K. G., Evans J. L., Alibali M. W., Saffran J. R. (2007). Can infants map meaning to newly segmented words? Statistical segmentation and word learning. *Psychological Science*.

[27] Bishop D. V. M., Brown B. B., Robson J. (1990). The relationship between phoneme discrimination, speech production, and language comprehension in cerebral-palsied individuals. *Journal of Speech and Hearing Research*.

[28] Bishop D. V., Robson J. (1989). Accurate non-word spelling despite congenital inability to speak: phoneme-grapheme conversion does not require subvocal articulation. *The British Journal of Psychology*.

[29] Bishop D. V., Robson J. (1989). Unimpaired short-term memory and rhyme judgement in congenitally speechless individuals: Implications for the notion of ‘articulatory coding’. *The Quarterly Journal of Experimental Psychology Section A*.

[30] Bishop D. V. M., Adams C. V., Norbury C. F. (2006). Distinct genetic influences on grammar and phonological short-term memory deficits: evidence from 6-year-old twins. *Genes, Brain and Behavior*.

[31] Krishnan S., Alcock K. J., Mercure E. (2013). Articulating novel words: children's oromotor skills predict non-word repetition abilities. *Journal of Speech, Language, and Hearing Research*.

[32] Goffman L., Smith A. (1999). Development and phonetic differentiation of speech movement patterns. *Journal of Experimental Psychology: Human Perception and Performance*.

[33] Green J. R., Moore C. A., Higashikawa M., Steeve R. W. (2000). The physiologic development of speech motor control: lip and jaw coordination. *Journal of Speech, Language, and Hearing Research*.

[34] Green J. R., Moore C. A., Reilly K. J. (2002). The sequential development of jaw and lip control for speech. *Journal of Speech, Language, and Hearing Research*.

[35] Grigos M. I., Patel R. (2007). Articulator movement associated with the development of prosodic control in children. *Journal of Speech, Language, and Hearing Research*.

[36] Grigos M. I., Patel R. (2010). Acquisition of articulatory control for sentential focus in children. *Journal of Phonetics*.

[37] Grigos M. I., Saxman J. H., Gordon A. M. (2005). Speech motor development during acquisition of the voicing contrast. *Journal of Speech, Language, and Hearing Research*.

[38] Sasisekaran J., Smith A., Sadagopan N., Weber-Fox C. (2010). Nonword repetition in children and adults: effects on movement coordination. *Developmental Science*.

[39] Smith A., Goffman L. (1998). Stability and patterning of speech movement sequences in children and adults. *Journal of Speech, Language, and Hearing Research*.

[40] Walsh B., Smith A. (2002). Articulatory movements in adolescents: evidence for protracted development of speech motor control processes. *Journal of Speech, Language, and Hearing Research*.

[41] Sharkey S. G., Folkins J. W. (1985). Variability of lip and jaw movements in children and adults: implications for the development of speech motor control. *Journal of Speech and Hearing Research*.

[42] Grigos M. I. (2009). Changes in articulator movement variability during phonemic development: a longitudinal study. *Journal of Speech, Language, and Hearing Research*.

[43] Smith A., Zelaznik H. N. (2004). Development of functional synergies for speech motor coordination in childhood and adolescence. *Developmental Psychobiology*.

[44] Maner K. J., Smith A., Grayson L. (2000). Influences of utterance length and complexity on speech motor performance in children and adults. *Journal of Speech, Language, and Hearing Research*.

[45] Dromey C., Benson A. (2003). Effects of concurrent motor, linguistic, or cognitive tasks on speech motor performance. *Journal of Speech, Language, and Hearing Research*.

[46] Walsh B., Smith A., Weber-Fox C. (2006). Short-term plasticity in children's speech motor systems. *Developmental Psychobiology*.

[47] McMillan C. T., Corley M., Lickley R. J. (2009). Articulatory evidence for feedback and competition in speech production. *Language and Cognitive Processes*.

[48] Goldrick M., Blumstein S. E. (2006). Cascading activation from phonological planning to articulatory processes: evidence from tongue twisters. *Language and Cognitive Processes*.

[49] Munson B. S., Solomon N. P. (2004). The effect of phonological neighborhood density on vowel articulation. *Journal of Speech, Language, and Hearing Research*.

[50] Baese-Berk M., Goldrick M. (2009). Mechanisms of interaction in speech production. *Language and Cognitive Processes*.

[51] Heisler L., Goffman L., Younger B. (2010). Lexical and articulatory interactions in children's language production. *Developmental Science*.

[52] Bishop D. V. M. (2006). Phonological short-term memory and syntactic impairment in specific language impairment. *Applied Psycholinguistics*.

[53] Bowey J. A. (2006). Clarifying the phonological processing account of nonword repetition. *Applied Psycholinguistics*.

[54] Service E. (2006). Phonological networks and new word learning. *Applied Psycholinguistics*.

[55] Gathercole S. E. (2006). Nonword repetition and word learning: the nature of the relationship. *Applied Psycholinguistics*.

[56] Smith A. (2006). Speech motor development: integrating muscles, movements, and linguistic units. *Journal of Communication Disorders*.

[57] Gathercole S. E., Willis C. S., Baddeley A. D., Emslie H. (1994). The children's test of nonword repetition: a test of phonological working memory. *Memory*.

[58] Goldman R., Fristoe M. (2002). *Goldman-Fristoe Test of Articulation*.

[59] Wiig E. H., Secord W., Semel E. (1992). *Clinical Evaluation of Language Fundamentals-Preschool*.

[60] Semel E., Wiig E. H., Secord W. A. (1995). *Clinical Evaluation of Language Fundamentals: CELF-3 Screening Test*.

[61] Hayden D., Square P. (1999). *VMPAC Manual*.

[62] Shriberg L. D., Kwiatkowski J. (1982). Phonological disorders III: a procedure for assessing severity of involvement. *Journal of Speech and Hearing Disorders*.

[64] (2007). *Matlab, Version 7.2 (Computer Software)*.

[65] Reuterskiöld-Wagner C., Sahlén B., Nyman A. (2005). Non-word repetition and non-word discrimination in Swedish preschool children. *Clinical Linguistics and Phonetics*.

[66] Vitevitch M. S. L., Luce P. A. (2004). A Web-based interface to calculate phonotactic probability for words and nonwords in English. *Behavior Research Methods, Instruments, and Computers*.

[67] Vitevitch M. S. (2006). Nonword repetition and language learning disorders. A developmental contingency framework. [Peer commentary on Gathercole, S. Nonword repetition and word learning: the nature of the relationship]. *Applied Psycholinguistics*.

[68] Smith A., Goffman L., Zelaznik H. N., Ying G., McGillem C. (1995). Spatiotemporal stability and patterning of speech movement sequences. *Experimental Brain Research*.

[69] Smith A., Johnson M., McGillem C., Goffman L. (2000). On the assessment of stability and patterning of speech movements. *Journal of Speech, Language, and Hearing Research*.

[70] Menyuk P., Looney P. L. (1972). A problem of language disorder: length versus structure. *Journal of Speech and Hearing Research*.

[71] Menyuk P., Looney P. L. (1972). Relationships among components of the grammar in language disorder. *Journal of Speech and Hearing Research*.

[72] Panagos J. M., Quine M. E., Klich R. J. (1979). Syntactic and phonological influences on children's articulation. *Journal of Speech and Hearing Research*.

[73] Panagos J. M., Prelock P. A. (1982). Phonological constraints on the sentence productions of language-disordered children. *Journal of Speech and Hearing Research*.

[74] Schmauch V. A., Panagos J. M., Klich R. J. (1978). Syntax influences the accuracy of consonant production in language-disordered children. *Journal of Communication Disorders*.

[75] Adams A.-M., Gathercole S. E. (2000). Limitations in working memory: implications for language development. *International Journal of Language and Communication Disorders*.

[76] Coady J. A., Evans J. L. (2008). Uses and interpretations of non-word repetition tasks in children with and without specific language impairments (SLI). *International Journal of Language and Communication Disorders*.

[77] Edwards J., Beckman M. E., Munson B. (2004). The interaction between vocabulary size and phonotactic probability effects on children’s production accuracy and fluency in nonword repetition. *Journal of Speech, Language, and Hearing Research*.

[78] Hulme C., Thomson N., Muir C., Lawrence A. (1984). Speech rate and the development of short-term memory span. *Journal of Experimental Child Psychology*.

[79] Hulme C., Tordoff V. (1989). Working memory development: the effects of speech rate, word length, and acoustic similarity on serial recall. *Journal of Experimental Child Psychology*.

